# Comparative Evaluation of Topical Stabilized Retinaldehyde 0.1% vs. 0.05% on Skin Biophysical and Biomechanical Parameters

**DOI:** 10.1111/srt.70326

**Published:** 2026-02-23

**Authors:** Anna Deda, Wiktoria Odrzywołek, Agata Lebiedowska, Anna Banyś, Małgorzata Bożek, Dagmara Kuca, Nina Wiśniewska, Dominika Wcisło‐Dziadecka, Sławomir Wilczyński

**Affiliations:** ^1^ Department of Practical Cosmetology and Skin Diagnostics Faculty of Pharmaceutical Sciences in Sosnowiec Medical University of Silesia in Katowice Sosnowiec Poland; ^2^ Department of Basic Biomedical Science Faculty of Pharmaceutical Sciences in Sosnowiec Medical University of Silesia in Katowice Sosnowiec Poland; ^3^ Department of Dermatology Military Institute of Medicine – National Research Institute Warsaw Poland

**Keywords:** antera 3d, cutometer, firmness, high‐frequency ultrasonography, retinaldehyde, skin texture, wrinkles

## Abstract

**Background:**

The aim of this study was to evaluate the biophysical and biomechanical effects of topical stabilized retinaldehyde (RAL) treatments on skin, using different concentrations of retinal (0.05% and 0.1%).

**Materials and Methods:**

Employing a split‐face design and noninvasive methods such as high‐frequency ultrasonography, the Antera 3D camera, and the Cutometer, the effectiveness of these preparations was assessed among 56 women aged 30–58 years.

**Results:**

The application of topical stabilized RAL resulted in statistically significant improvements in skin parameters. High‐frequency ultrasonography revealed increased dermal density across all facial regions after 24 weeks, while Cutometer measurements showed enhanced skin elasticity and viscoelasticity. Antera 3D imaging demonstrated significant reductions in wrinkle parameters, particularly with the higher concentration (0.1%) of RAL. The treatments were generally well‐tolerated, with minimal adverse effects reported, highlighting their suitability for sensitive skin.

**Conclusion:**

Results indicated significant improvements in skin texture and firmness, particularly with the higher 0.1% concentration of RAL over 24‐week period, making it a promising option for long‐term skin rejuvenation therapy.

## 1 Introduction

Retinoids, a diverse class of compounds derived from vitamin A, are extensively used in dermatological treatments and skin care. These compounds are systematically classified into four generations based on their molecular configurations and receptor targeting properties [1]. The first category comprises natural compounds that contain a nonaromatic β‐ionone fragment within their structure. Included in this category are retinol, retinaldehyde (RAL), and retinoic acid [2]. While their effects on seborrhea reduction are moderate, retinoids are particularly effective in promoting comedolysis and normalizing the differentiation and proliferation of keratinocytes [3, 4]. RAL is recognized for its potent antiaging capabilities, particularly its rapid action in skin rejuvenation due to its proximity to retinoic acid in the vitamin A metabolic pathway [5]. RAL is the immediate precursor of retinoic acid. In this study, we used a stabilized retinaldehyde cyclodextrin complex prepared by the reaction of RAL (polyene aldehyde) with cyclodextrin and hydrolyzed glycosaminoglycans that enhances penetration into the skin layers.

High‐frequency ultrasound (HFUS) is particularly noted for its ability to provide detailed images of the epidermis and deeper skin layers. This technology, utilizing frequencies of 50 MHz, enables visualization of skin structures with high resolution, which is beneficial for assessing skin thickness, density, and even the morphology of deeper skin layers [6]. It can detect features such as increased collagen production, which often correlates with echogenicity in ultrasound images [7]. The Cutometer is another well‐established instrument used extensively to assess the biomechanical properties of the skin. It measures elasticity and firmness by applying suction and observing the skin's ability to return to its original state after deformation. This method is critical for evaluating the effectiveness of skincare products and treatments that aim to improve skin elasticity and firmness, and it is also utilized in clinical practice validation [8]. Lastly, the Antera 3D camera offers advanced capabilities in skin analysis by providing high‐resolution 3D images. This can be particularly useful for a detailed assessment of surface topography, including wrinkle analysis and skin texture improvements over time [9]. It provides quantifiable data on skin roughness and wrinkle depth, making it an excellent tool for before‐and‐after studies of cosmetic and antiaging treatments. By integrating these methods, this study not only leverages their individual strengths but also provides a holistic view of how skin responds to treatments at both the surface and subsurface levels.

## 2 Materials and Methods

### 2.1 Patients

Fifty‐six women (mean age: 44.25 ± 8.1 years) were enrolled in the study. Only female subjects were included in this study as they represent the primary target population for antiaging cosmetic treatments and typically demonstrate higher compliance with skincare regimens. One participant withdrew from the study due to the development of facial erythema and significant discomfort. The reduced sample size in specific analyses (denoted by *N*) was due to participant absence on scheduled measurement dates, technical measurement failures, or incomplete data registration.

The inclusion criteria for the study were as follows:

- Age 30–60 years
- Presence of wrinkles, dark spots, acne, seborrhea
- Decreased skin firmness

The exclusion criteria were:

- Exacerbated active atopic dermatitis on the face
- Pregnancy
- Skin infections
- Numerous erosions and excoriations on facial skin
- No aesthetic medical treatments within the preceding 6 months
- Administration of botulinum toxin within the preceding 3 months
- Irritated, dry skin.

### 2.2 The Course of the Study

This study employed a split‐face blinded design where each participant served as their own control, minimizing inter‐individual variability. Two products containing 0.05% RAL and 0.1% RAL were tested. Volunteers received cosmetic packages labeled “left” and “right” and were instructed to use them on the corresponding sides of the face without knowing which concentration was assigned to which side, ensuring participant blinding. The study protocol assigned the 0.1% RAL preparation to the left side of the face and the 0.05% RAL preparation to the right side of the face.

### 2.3 INCI Composition

INCI composition of the product applied to the left side of the face: Aqua, C15‐19 Alkane, Squalane, Glycerin, Caprylic/Capric Triglyceride, Tripelargonin, Pentylene Glycol, Arachidyl Alcohol, Niacinamide, Cyclodextrin, Cetyl Alcohol, Behenyl Alcohol, Propanediol, Hydrolyzed Glycosaminoglycans, Diglucosyl Gallic Acid, *Lactobacillus*/Soybean Ferment Extract, Madecassic Acid, Tranexamic Acid, Saccharomyces/Viscum Album Ferment Extract, Retinal, Rubus Chamaemorus Seed Oil, Asiaticoside, *Saccharomyces*/*Imperata cylindrica* Root Ferment Extract, Biosaccharide Gum‐1, *Lonicera caprifolium* Flower Extract, Asiatic Acid, *Lonicera japonica* Flower Extract, Tocopheryl Acetate, Mannitol, Cetearyl Olivate, Arachidyl Glucoside, Coco Caprylate/Caprate, Sorbitan Olivate, Phosphatidylcholine, Decyl Glucoside, Hydroxyethyl Acrylate/Sodium Acryloyldimethyl Taurate Copolymer, Xanthan Gum, Sodium Levulinate, Glyceryl Caprylate, Sodium Chloride, Sodium Phytate, Sorbitan Isostearate, Polysorbate 60, Sodium Anisate, Potassium Sorbate, Sodium Benzoate, and Hydroxyacetophenone.

INCI composition of the product applied to the right side of the face: Aqua, Glycerin, C15‐19 Alkane, Polyglyceryl‐6 Stearate, Caprylic/Capric Triglyceride, Pentylene Glycol, Acacia Senegal Gum, Squalane, Butylene Glycol, Hydrolyzed Glycosaminoglycans, Glyceryl Stearate, Cetearyl Alcohol, *Prunus amygdalus* Dulcis Oil, *Macadamia ternifolia* Seed Oil, *Butyrospermum parkii* Butter, *Rubus chamaemorus* Seed Oil, Madecassic Acid, Glycine, Crocus Chrysanthus Bulb Extract, *Lonicera caprifolium* Flower Extract, *Lonicera japonica* Flower Extract, Retinal, Asiaticoside, Serine, Glutamic Acid, Asiatic Acid, Aspartic Acid, Leucine, Alanine, Lysine, Isoleucine, Histidine, Arginine, Tocopheryl Acetate, Tyrosine, Phenylalanine, Valine, Threonine, Proline, Tocopherol, Sodium Hyaluronate, Cyclodextrin, Coco Caprylate/Caprate, Olus Oil, Sodium Phytate, Sorbitan Isostearate, Polysorbate 60, 1,2‐Hexanediol, Caprylyl Glycol, Polyglyceryl‐6 Behenate, Hydroxyethyl Acrylate/Sodium Acryloyldimethyl Taurate Copolymer, Xanthan Gum, and Hydroxyacetophenone.

### 2.4 Application Protocol

Participants applied approximately 0.5 mL (corresponding to a pea‐sized amount) of each preparation to the corresponding side of the face in the evening. The application frequency started with twice weekly for the first 2 weeks, then gradually increased to three times per week in the third week and four times per week in the fourth week based on skin tolerance. Each product was applied after cleansing and drying the face, avoiding the eye and mouth areas.

To eliminate the influence of other skincare products used by the volunteers on the test results, all volunteers were instructed to use the same skincare regimen throughout the study period, which included Ph.Doctor lipid face cream and Ph.Doctor soft facial cleansing lotion. Additionally, to ensure consistent protection against UV radiation, all volunteers used Pharmaceris SPF 50 sunscreen throughout the entire duration of the study.

### 2.5 Adverse Events Monitoring

Adverse events were monitored using both active (clinical evaluation and structured questioning at each visit) and passive (participant self‐reporting between visits) methods. All events were documented and categorized according to standard dermatological criteria.

### 2.6 Measurements

To monitor the effects of therapy, evaluations were conducted at the following time points:

- T0 – assessment of skin parameters before starting therapy,
- T1 – assessment of skin parameters after 12 weeks of therapy,
- T2 – assessment of skin parameters after 24 weeks of therapy.

Measurements were performed in a controlled environment with constant room temperature (22  ±  1°C) and relative humidity (45%–50%).

The study employed Antera 3D camera (Miravex Limited, Ireland) to capture and analyze skin condition across different facial regions (forehead, eyes, cheeks, and chin on both sides of the face). All Antera 3D images were taken in a seated position, with standardized lighting conditions and consistent camera‐to‐skin distance. Antera 3D allows for the measurement of various features associated with wrinkles. A filter was applied to the reconstructed 3D skin surface to distinguish the curvatures characteristic of wrinkles (i.e., small, fine depressions) from the normalized reference surface (representing the skin surface in the absence of wrinkles). The normalized reference shape was then subtracted to isolate the wrinkles themselves.

DUB SkinScanner, a high‐frequency ultrasound system, equipped with a 50 MHz head, was utilized to assess skin density (echo intensity) and epidermal thickness (entry echo) in B‐scan projection. Epidermal thickness was calculated in millimeters based on the A‐scan. Skin density was measured directly beneath the epidermis using the ‘region of interest’ (ROI) function in the DUB SkinScanner software (DUB‐SkinScanner75 5.21.). Measurements were consistently taken at predetermined locations, and all images were acquired using standardized parameters. To standardize measurements across sessions and participants, predefined facial zones were mapped using anatomical landmarks and marked on clinical photographs taken at baseline. This facial mapping allowed precise re‐identification of measurement sites during follow‐up visits. Although the scans were not performed by a single operator, all personnel underwent the same training and followed a detailed imaging protocol. Prior to measurements, ultrasonic gel was applied to the skin surface. Images were recorded perpendicular to the skin surface with minimal pressure from the operator. The system utilizes a unique water‐coupled probe placed in a measurement chamber filled with distilled water. Unlike standard ultrasound transducers, the DUB SkinScanner's probe does not come into direct contact with the skin. Instead, the ultrasound waves are transmitted through water, and the probe moves freely within the water chamber. This design eliminates any mechanical pressure on the measured skin, thereby reducing the risk of compression artifacts and enhancing reproducibility.

The Cutometer MPA 580 (Courage+Khazaka, Köln, Germany) was utilized to evaluate skin viscoelasticity. Following application of the probe to the skin, the device applies negative pressure to create suction. As the skin is lifted, the device records changes in position and deformation, enabling assessment of skin elasticity and firmness. Measurements were performed under constant vacuum conditions, and skin deformation curves derived from these measurements aid in evaluating the skin's biomechanical properties

The following parameters were extracted from the deformation curves to quantify skin elasticity:

R2 (Ua/Uf)—Gross elasticity: Represents the overall ability of the skin to return to its original state after deformation. It is calculated as the ratio of immediate recovery (Ua) to the maximum deformation (Uf). Values closer to 1 (100%) indicate better global elasticity.

R5 (Ur/Ue)—Net elasticity: Describes the purely elastic portion of the skin's response, excluding viscous deformation. It is calculated as the ratio of immediate recovery (Ur) to immediate deformation (Ue). Higher values indicate more elastic and less viscoelastic behavior.

R7 (Ur/Uf)—Elastic recovery ratio: Measures the proportion of elastic recovery (Ur) relative to total deformation (Uf). It reflects the dominance of elastic behavior in the overall skin response. Higher values indicate a more elastic skin profile.

Q1 – Elastic recovery: Quantifies the rapid, purely elastic recoil of the skin immediately after suction release. A higher Q1 value corresponds to greater skin elasticity.

Q2 – Viscoelastic recovery: Reflects the combined contribution of both elastic and delayed (viscous) recovery of the skin. Higher values indicate better viscoelastic behavior and overall skin resilience.

All measurements were performed using a 2 mm probe aperture, and each test was conducted under standardized environmental and technical conditions.

### 2.7 Statistical Analysis

All statistical analyses were conducted using Statistica 13.3 software (TIBCO Software, Palo Alto, CA, USA). Given the lack of normal distribution for a significant number of evaluated parameters, as indicated by the Shapiro–Wilk test, all variables were analyzed using nonparametric tests. Box plots were utilized for appropriate visualization, depicting the median and interquartile range.

For the analysis of variables at time points T0, T1, and T2, the Friedman ANOVA with a dedicated post‐hoc test was applied. When assessing the relationship between the results obtained at times T0 and T1, as well as when comparing the effects of treatment on the left and right sides of the face, the Wilcoxon signed‐rank test was utilized. Statistical significance was determined at *p* < 0.05, while *p* values within the range of 0.05–0.06 indicated a tendency toward significance.

## 3 Results

### 3.1 Antera 3D Imaging

**FIGURE 1:**
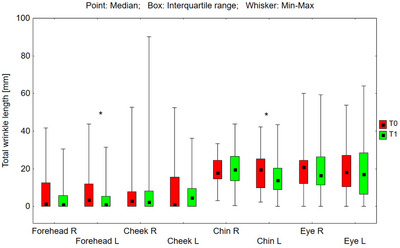
Total length of wrinkles (mm) measured on the right (P) and left (L) side of the face on the forehead, cheeks, chin, and eye area before application of the preparation (T0) and after 12 weeks (T1) of its use (Wilcoxon signed‐rank test, *p < 0.05, nz‐ not significant) (*N* = 54).

**FIGURE 2:**
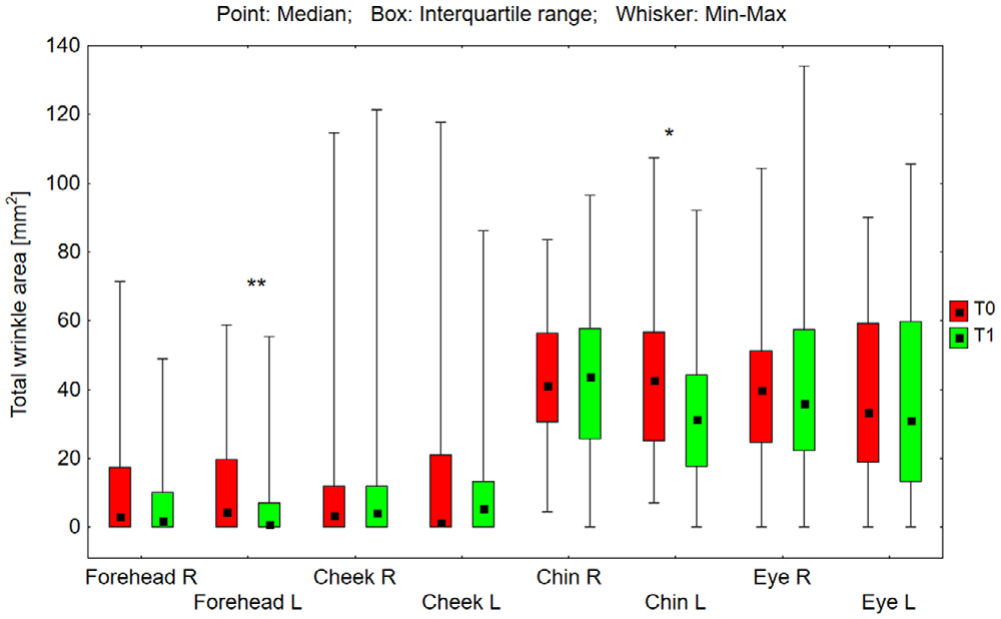
Total wrinkle area (mm^2^) measured on the right (P) and left (L) side of the face on the forehead, cheeks, chin, and eye area before application of the preparation (T0) and after 12 weeks (T1) of its use (Wilcoxon signed‐rank test, *p < 0.05, **p < 0.01, ns‐not significant) (*N* = 54).

**FIGURE 3:**
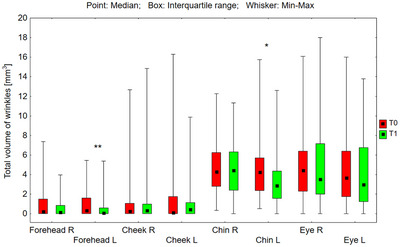
Total volume of wrinkles (mm^3^) measured on the right (P) and left (L) side of the face on the forehead, cheeks, chin, and eye area before application of the preparation (T0) and after 12 weeks (T1) of its use (Wilcoxon signed‐rank test, *p < 0.05, **p < 0.01, ns‐not significant) (*N* = 54).

**FIGURE 4:**
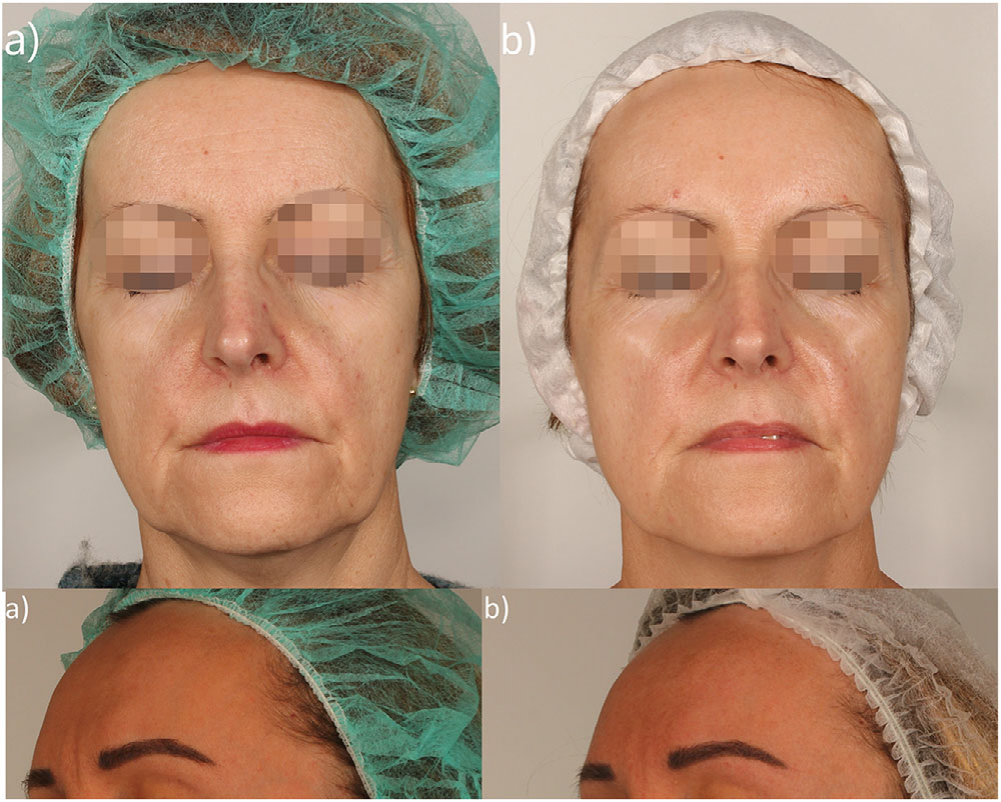
Photograph of volunteers (a) before and (b) after 24 weeks of using the tested preparations. Eyes have been pixelated to protect participant privacy.

The tested preparations had a statistically significant effect on reducing wrinkle parameters in areas where a higher concentration (0.1%) of RAL was applied (Figures 1, 2, 3, 4).

On the left forehead, the median total wrinkle length in the marked area decreased from 3.57 to 1.03 mm (by 71.1%), and the upper quartile from 11.97 to 5.43 mm (by 54.6%), and these were statistically significant differences (*p* = 0.018) (Figure 1). On the left side of the chin, the median total length of wrinkles in the marked area decreased from 19.53 to 13.97 mm (by 28.5%), and the upper quartile from 25.27 to 20.34 mm (by 19.5%), representing statistically significant differences (*p* = 0.040). No statistically significant changes were observed on the right side of the face. When comparing the differences in total wrinkle length resulting from treatment (T1–T0) on the left versus the right side of the forehead, the result did not reach statistical significance (*p* = 0.946). However, statistical analysis of the treatment‐induced changes (T1–T0) in total wrinkle length on the left and right sides of the chin revealed that the reduction observed on the left side was significantly greater than that on the right side (*p* = 0.031).

On the left side of the forehead, the median of the total wrinkle area in the marked area decreased from 4.53 to 0.91 mm^2^ (by 79.9%), and the upper quartile from 19.84 to 7.06 mm^2^ (by 64.4%), and these were statistically significant differences (*p* = 0.009) (Figure 2). On the left side of the chin, the median of the total wrinkle area in the marked area decreased from 42.86 to 31.27 mm^2^ (by 27.0%), and the upper quartile from 56.71 to 44.26 mm^2^ (by 22.0%), and these were statistically significant differences (*p* = 0.015). When comparing the differences in total wrinkle area resulting from treatment (T1–T0) on the left and right sides of the forehead, the result did not reach statistical significance (*p* = 0.492). However, statistical analysis of the treatment‐induced changes (T1–T0) in total wrinkle area on the left and right sides of the chin demonstrated that the reduction observed on the left side was significantly greater than that on the right side (*p* = 0.033).

On the left side of the forehead, the median of the total volume of wrinkles in the marked area decreased from 0.32 to 0.06 mm^3^ (by 81.25%), and the upper quartile from 1.62 to 0.61 mm^3^ (by 62.3%), and these were statistically significant differences (*p* = 0.008) (Figure 3). On the left side of the chin, the median of the total volume of wrinkles in the marked area decreased from 4.25 to 2.87 mm^3^ (by 32.5%), and the upper quartile from 5.70 to 4.37 mm^3^ (by 23.3%), and these were statistically significant differences (*p* = 0.024). The treatment‐induced differences (T1–T0) in total wrinkle volume calculated for the left and right sides of the face were not statistically significant for the forehead (*p* = 0.386) or the chin (*p* = 0.115).

### 3.2 High Frequency Ultrasound

**FIGURE 5:**
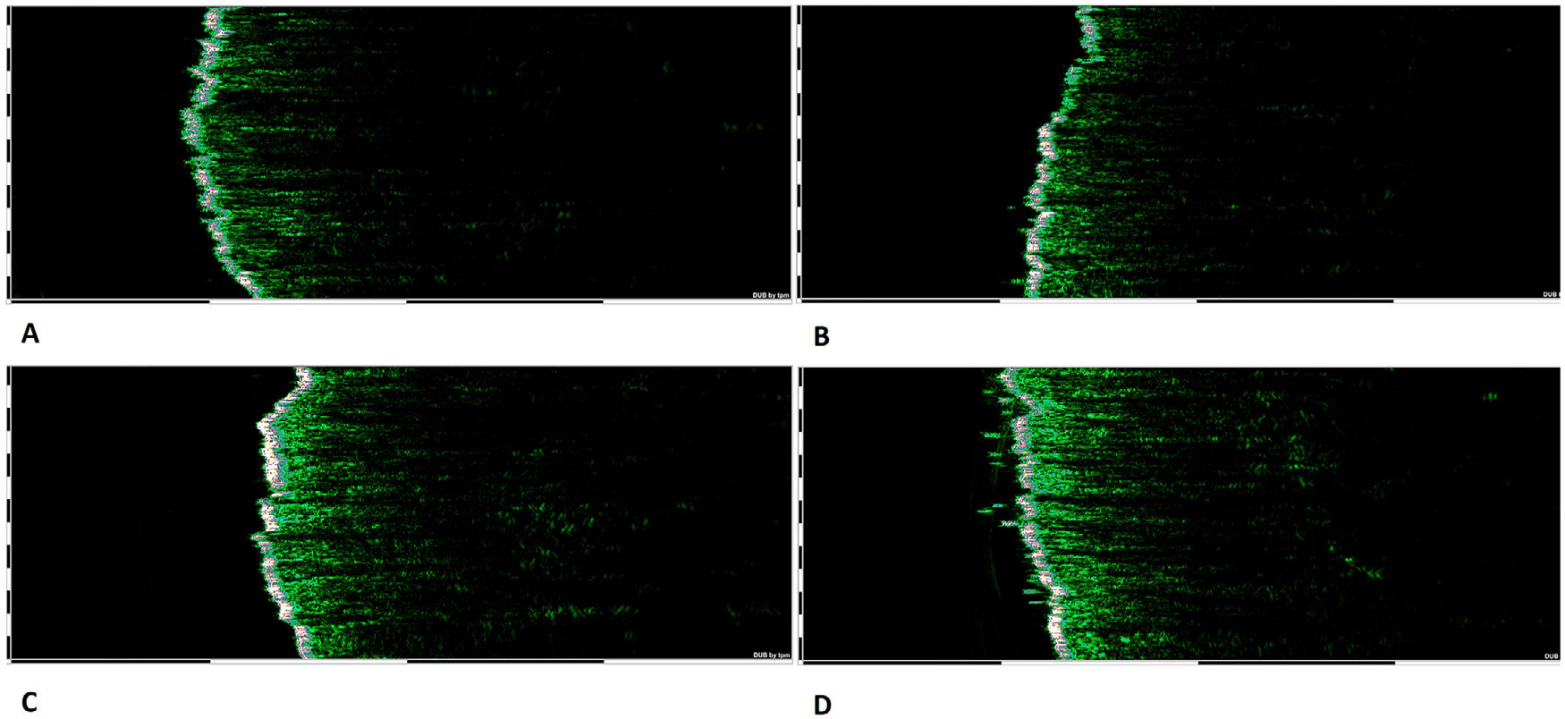
Example ultrasound scans for an example volunteer showing: (a) left side of the chin before application of the tested preparation, (b) right side of the chin before application of the tested preparation; (c) left side of the chin after application of the tested preparations, (d) right side of the chin after application of the tested preparations.

Figure 5 shows sample ultrasound scans before and after the use of the tested preparations.

#### 3.2.1 Analysis of Epidermal Thickness

**FIGURE 6:**
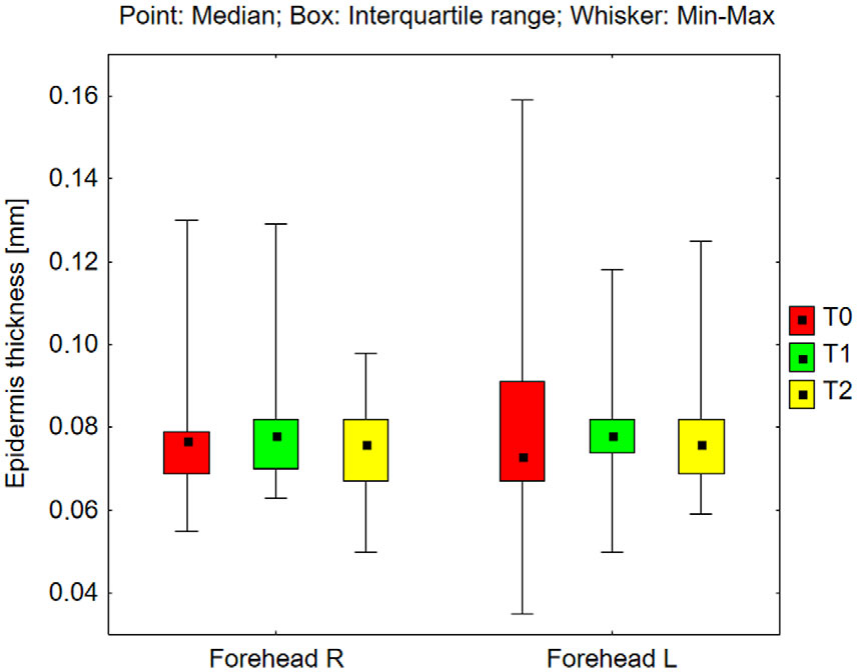
Epidermis thickness measured on the right and left side of the forehead before application of the preparation (T0), and after 12 weeks (T1), and 24 weeks (T2) of its use (Friedman's Anova, n.s—not significant).

**FIGURE 7:**
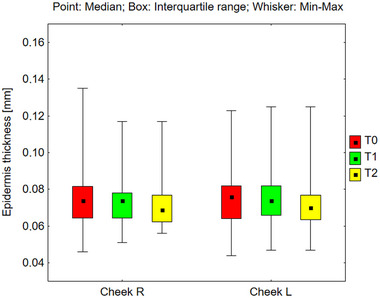
Epidermis thickness measured on the right and left cheek before application of the preparation (T0), and after 12 weeks (T1), and 24 weeks (T2) of its use (Friedman's Anova, n.s—not significant).

**FIGURE 8:**
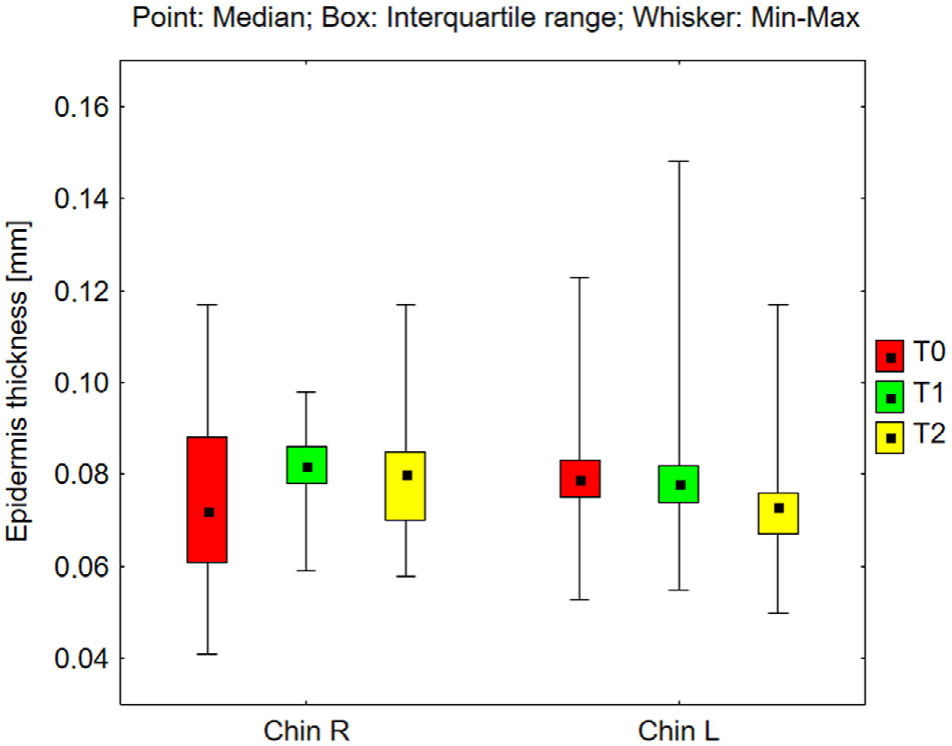
Epidermis thickness measured on the right and left side of the chin before application of the preparation (T0), and after 12 weeks (T1), and 24 weeks (T2) of its use (Friedman's Anova, n.s—not significant).

Epidermal thickness was measured across various facial regions, including the right and left sides of the forehead, cheeks, and chin (Figures 6, 7, 8). However, no statistically significant changes were observed across the different time periods under the influence of the preparation. The absence of alterations in total epidermal thickness following the application of the tested products suggests that RAL did not cause significant structural changes in the epidermis as measured by high‐frequency ultrasound. However, this measurement reflects total epidermal thickness and does not provide specific information about individual epidermal layers, including the stratum corneum.

#### 3.2.2 Analysis of Dermal Density

**FIGURE 9:**
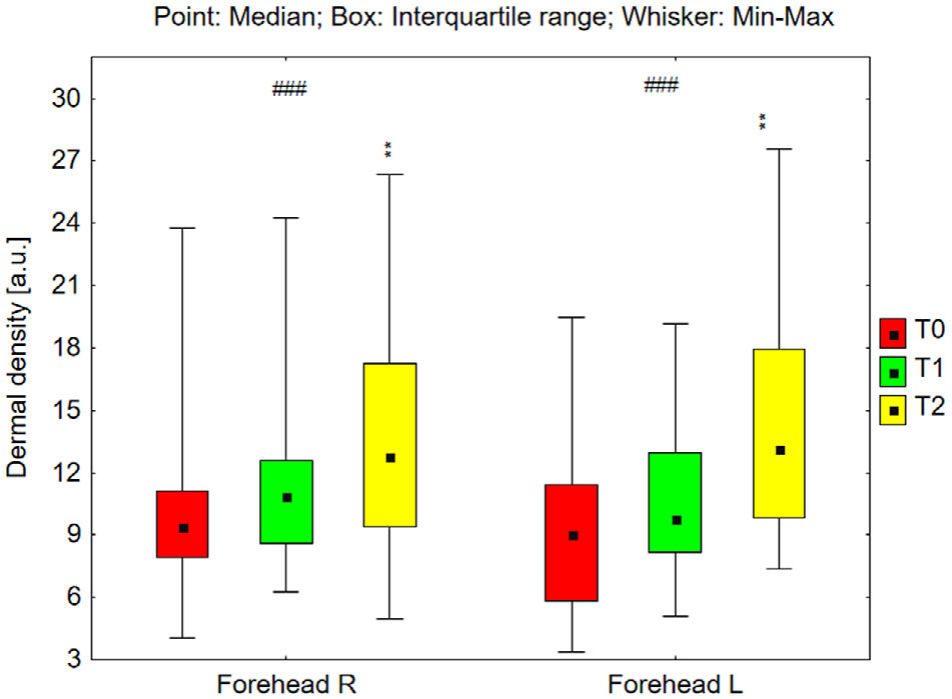
The dermal density measured on both sides of the forehead before the application of the preparation (T0), and after 12 weeks (T1), and 24 weeks (T2) of its use (Friedman's ANOVA ###*p* < 0.001; post‐hoc *p < 0.05, Right: T0 vs. T2, T1 vs. T2, Left: T0 vs. T2, T1 vs. T2).

**FIGURE 10:**
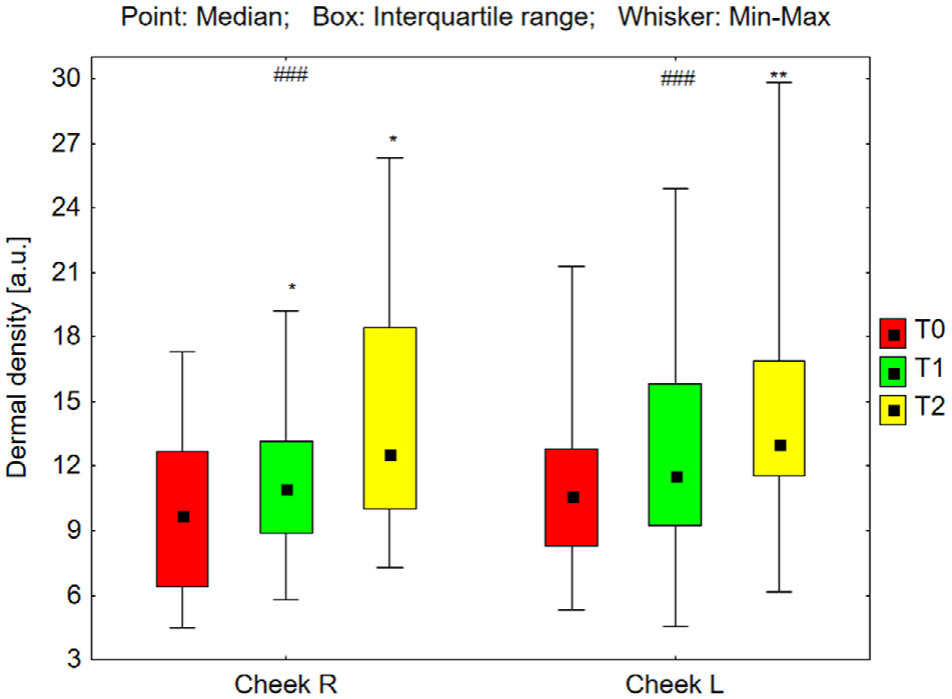
The dermal density measured on the right and left cheek before the application of the preparation (T0), and after 12 weeks (T1), and 24 weeks (T2) of its use (Friedman's ANOVA ###*p* < 0.001; post‐hoc *p < 0.05, Right: T0 vs. T2, T1 vs. T2, Left: T0 vs. T2, T1 vs. T2).

**FIGURE 11:**
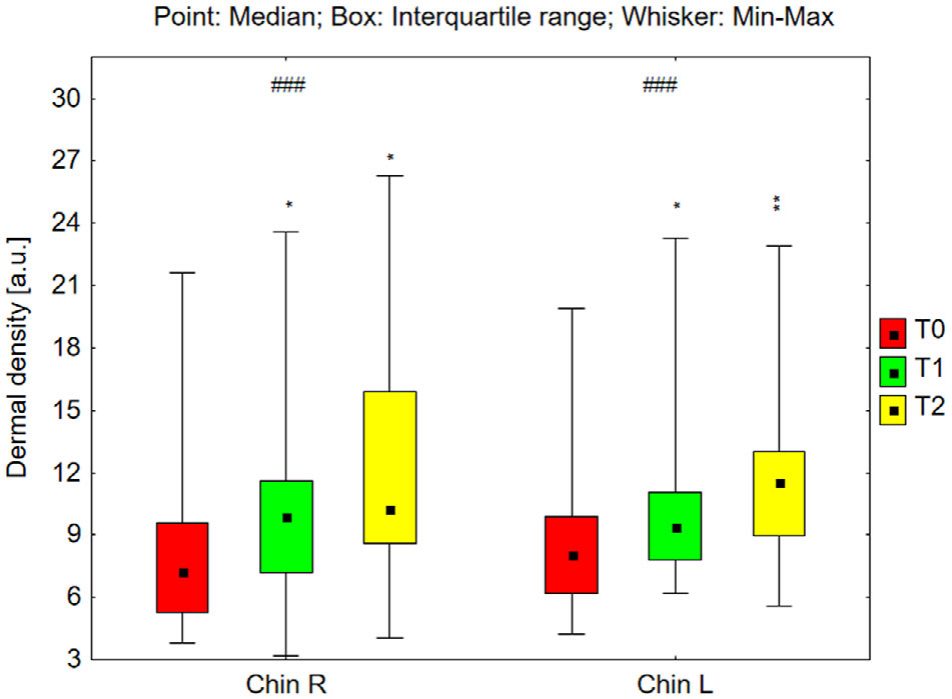
The dermal density measured on both sides of the before the chin application of the preparation (T0), and after 12 weeks (T1), and 24 weeks (T2) of its use (Friedman's ANOVA ###*p* < 0.001; post‐hoc *p < 0.05, Right: T0 vs. T2, T1 vs. T2, Left: T0 vs. T2, T1 vs. T2).

Dermal density was assessed across various facial regions: the forehead, cheeks, and chin (Figures 9, 10, 11). Statistically significant increases in dermal density were observed bilaterally on the forehead (*p* < 0.001), cheeks (*p* < 0.001), and chin (*p* < 0.001) under the influence of the preparation. Post‐hoc analyses revealed increased dermal density after 24 weeks of using the preparation (T2) compared to the skin density before its use (T0), and compared to the skin density after 12 weeks of its use (T1). This trend exhibited consistency across all examined areas. The median values of dermal density for each region are presented as follows: on the right side of the forehead 9.41 (T0), 10.84 (T1), and 12.76 (T2); on the left side of the forehead, 9.05 (T0), 9.79 (T1), and 13.16 (T2). Similarly, on the right cheek, the values were 9.71 (T0), 10.93 (T1), and 12.54 (T2), and on the left cheek, 10.58 (T0), 11.53 (T1), and 13.05 (T2). On the right side of the chin, the values were 7.26 (T0), 9.87 (T1), and 10.24 (T2), and on the left side, 8.03 (T0), 9.37 (T1), and 11.55 (T2). The increase in median dermal density at T1 and T2 compared to T0 was as follows: on the right side of the forehead by 15.2% at T1 and 35.5% at T2, on the left side of the forehead by 8.2% at T1 and 45.4% at T2, on the right cheek by 7.0% at T1 and 29.1% at T2, on the left cheek by 9.0% at T1 and 23.3% at T2, on the right side of the chin by 36.0% at T1 and 41.9% at T2, and on the left side of the chin by 16.7% at T1 and 43.8% at T2.

The treatment‐induced differences in dermal density (T1–T0 and T2–T0) calculated for the left and right sides of the face did not differ significantly in any of the examined areas: forehead (T1–T0; *p* = 0.879, T2–T0; *p* = 0.649), cheeks (T1–T0; *p* = 0.940, T2–T0; *p* = 0.537), and chin (T1–T0; *p* = 0.159, T2–T0; *p* = 0.102).

The observed increase in skin density across bilateral forehead, cheeks, and chin regions is attributed to increased echogenicity, suggesting both the formation of new collagen fibers and thickening of existing ones. Posttreatment ultrasound examinations revealed increased hyperechoic reflections originating from collagen fibers. Higher collagen fiber density corresponds to increased ultrasound reflection, resulting in a brighter image. Therefore, it can be concluded that the tested products exerted a positive effect on skin density, thus contributing to improved skin condition. An increase in skin density was observed just 12 weeks after starting to use the preparations, across both concentrations. Furthermore, between weeks 12 and 24 of treatment, a statistically significant surge in skin density was observed in the analyzed regions, indicating that a 6‐month treatment regimen was more effective than a 3‐month regimen.

### 3.3 Cutometer

#### 3.3.1 R2 Parameter

**FIGURE 12:**
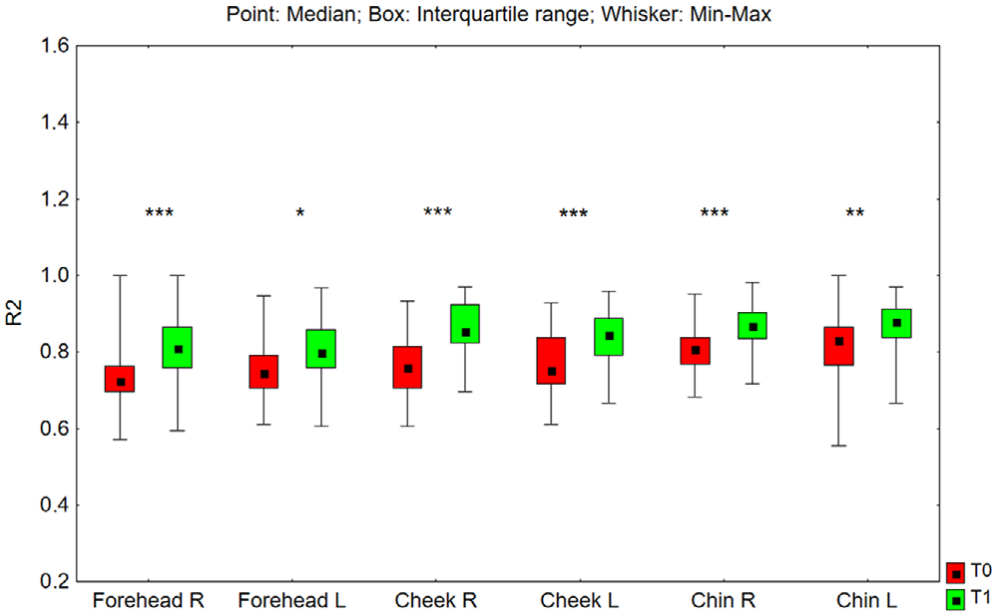
The R2 parameter measured on the right (P) and left (L) side of the face on the forehead, cheeks, and chin before application of the preparation (T0) and after 24 weeks (T1) of its use (Wilcoxon signed‐rank test, *p < 0.05, **p < 0.01, ***p < 0.001) (*N* = 52).

R2 reflects the skin's capacity to revert to its initial state following deformation. The value of the R2 parameter after 24 weeks of using the preparation increased statistically significantly in all tested facial zones: on the right side of the forehead (*p* < 0.001), on the left side of the forehead (*p* = 0.010), on the right cheek (*p* < 0.001), on the left cheek (*p* < 0.001), right side of the chin (*p* < 0.001), and left side of the chin (*p* = 0.002) (Figure 12). The median value of the R2 parameter increased in the examined zones as follows: on the right side of the forehead from 0.725 to 0.809 (by 11.6%), on the left side of the forehead from 0.745 to 0.799 (by 7.2%), on the right cheek from 0.758 to 0.855 (by 12.8%), on the left cheek from 0.751 to 0.844 (by 12.4%), on the right side chin from 0.807 to 0.868 (by 7.6%), and on the left side of the chin from 0.830 to 0.880 (by 6.0%).

The comparison of treatment‐induced changes (T1–T0) in the R2 parameter between the left and right sides of the face showed no statistically significant differences for the forehead (*p* = 0.136) and chin (*p* = 0.317). However, for the cheeks, the differences between the two sides were statistically significant (*p* = 0.021).

#### 3.3.2 R5 Parameter

**FIGURE 13:**
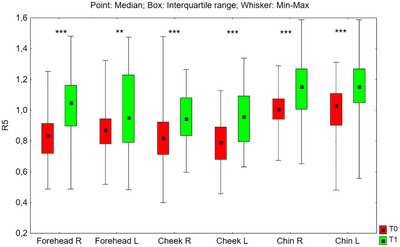
The R5 parameter measured on the right (P) and left (L) side of the face on the forehead, cheeks, and chin before application of the preparation (T0) and after 24 weeks (T1) of its use (Wilcoxon signed‐rank test, *p < 0.05, **p < 0.01, ***p < 0.001) (*N* = 52).

The value of the R5 parameter after 24 weeks of using the preparation increased statistically significantly in all tested facial zones: on the right side of the forehead (*p* < 0.001), on the left side of the forehead (*p* = 0.004), on the right cheek (*p* < 0.001), left cheek (*p* < 0.001), on the right side of the chin (*p* < 0.001), and on the left side of the chin (*p* < 0.001) (Figure 13). The median value of the R5 parameter increased in the studied zones as follows: on the right side of the forehead from 0.834 to 1.048 (by 25.7%), on the left side of the forehead from 0.870 to 0.951 (by 9.3%), on the right cheek from 0.820 to 0.944 (by 15.1%), on the left cheek from 0.791 to 0.959 (by 21.2%), on the right‐side chin from 1.006 to 1.154 (by 14.7%), and on the left side of the chin from 1.031 to 1.154 (by 11.9%).

The comparison of treatment‐induced changes (T1–T0) in the R5 parameter between the left and right sides of the face showed no statistically significant differences for the forehead (*p* = 0.630), cheeks (*p* = 0.874), or chin (*p* = 0.589).

#### 3.3.3 R7 Parameter

**FIGURE 14:**
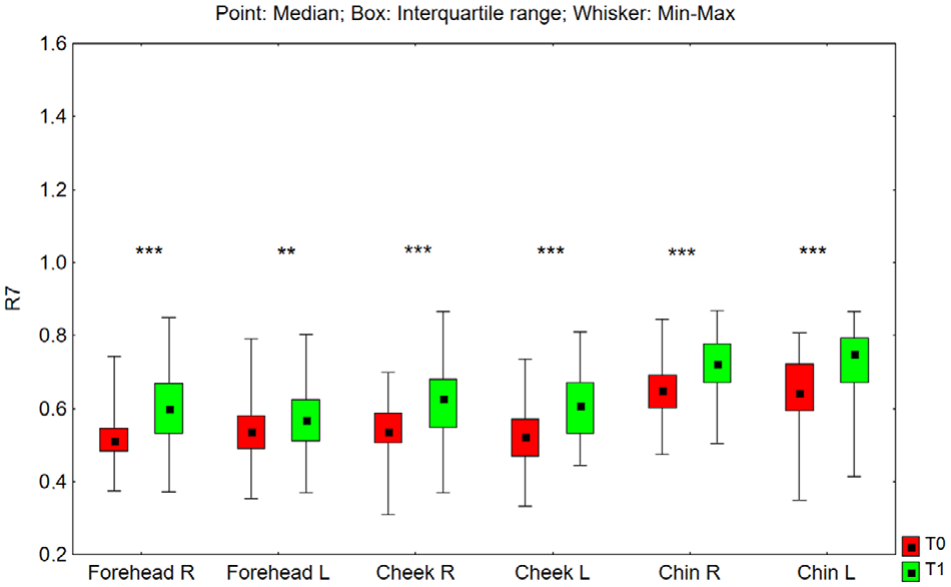
The R7 parameter measured on the right (P) and left (L) side of the face on the forehead, cheeks, and chin before application of the preparation (T0) and after 24 weeks (T1) of its use (Wilcoxon signed‐rank test, *p < 0.05, **p < 0.01, ***p < 0.001) (*N* = 52).

The value of the R7 parameter after 24 weeks of using the preparation increased statistically significantly in all tested facial zones: on the right side of the forehead (*p* < 0.001), on the left side of the forehead (*p* = 0.007), on the right cheek (*p* < 0.001), on the left cheek (*p* < 0.001), on the right side of the chin (*p* < 0.001), and on the left side of the chin (*p* < 0.001) (Figure 14). The median value of the R7 parameter increased in the studied zones as follows: on the right side of the forehead from 0.512 to 0.600 (by 17.2%), on the left side of the forehead from 0.537 to 0.569 (by 6.0%), on the right cheek from 0.537 to 0.627 (by 16.8%), on the left cheek from 0.524 to 0.609 (by 16.2%), on the right‐side chin from 0.651 to 0.721 (by 10.8%), and on the left side of the chin from 0.643 to 0.750 (by 16.6%).

The comparison of treatment‐induced changes (T1–T0) in the R7 parameter between the left and right sides of the face showed no statistically significant differences for the cheeks (*p* = 0.136) or chin (*p* = 0.317). However, for the forehead, the differences between the two sides were statistically significant (*p* = 0.018).

#### 3.3.4 Q1 Parameter

**FIGURE 15:**
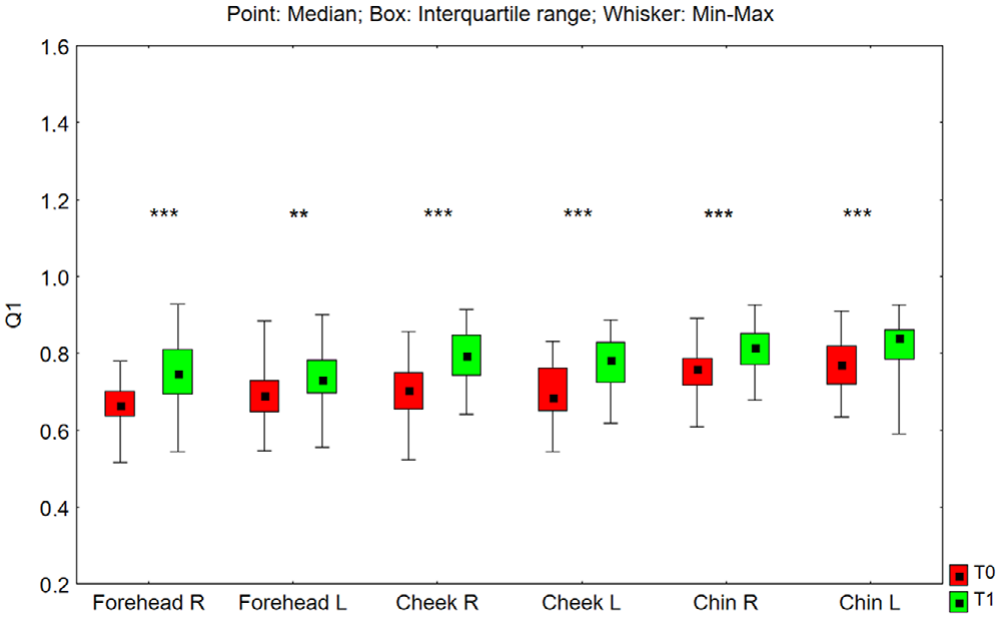
The Q1 parameter measured on the right (P) and left (L) side of the face on the forehead, cheeks, and chin before application of the preparation (T0) and after 24 weeks (T1) of its use (Wilcoxon signed‐rank test, *p < 0.05, **p < 0.01, ***p < 0.001) (*N* = 52).

The value of the Q1 parameter after 24 weeks of using the preparation increased statistically significantly in all tested facial zones: on the right side of the forehead (*p* < 0.001), on the left side of the forehead (*p* = 0.006), on the right cheek (*p* < 0.001), left cheek (*p* < 0.001), on the right side of the chin (*p* < 0.001), and on the left side of the chin (*p* < 0.001) (Figure 15). The median value of the Q1 parameter increased in the examined zones as follows: on the right side of the forehead from 0.663 to 0.749 (by 13.0%), on the left side of the forehead from 0.690 to 0.730 (by 5.8%), on the right cheek from 0.704 to 0.792 (by 12.5%), on the left cheek from 0.685 to 0.782 (by 14.2%), on the right‐side chin from 0.759 to 0.815 (by 7.4%), and on the left side of the chin from 0.771 to 0.839 (by 8.8%).

The comparison of treatment‐induced changes (T1–T0) in the Q1 parameter between the left and right sides of the face showed no statistically significant differences for the forehead (*p* = 0.089), cheeks (*p* = 0.097), or chin (*p* = 0.569).

#### 3.3.5 Q2 Parameter

**FIGURE 16:**
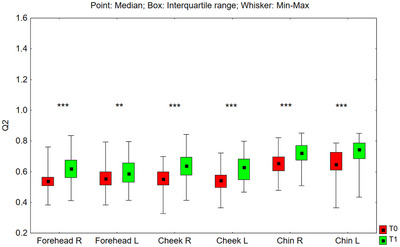
The Q2 parameter measured on the right (P) and left (L) side of the face on the forehead, cheeks, and chin before application of the preparation (T0) and after 24 weeks (T1) of its use (Wilcoxon signed‐rank test, *p < 0.05, **p < 0.01, ***p < 0.001) (*N* = 52).

The value of the Q2 parameter after 24 weeks of using the preparation increased statistically significantly in all tested facial zones: on the right side of the forehead (*p* < 0.001), on the left side of the forehead (*p* = 0.005), on the right cheek (*p* < 0.001), on the left cheek (*p* < 0.001), on the right side of the chin (*p* < 0.001) and on the left side of the chin (*p* < 0.001) (Figure 16). The median value of the Q2 parameter increased in the studied zones as follows: on the right side of the forehead from 0.539 to 0.620 (by 15.0%), on the left side of the forehead from 0.554 to 0.588 (by 6.1%), on the right cheek from 0.533 to 0.640 (by 20.1%), on the left cheek from 0.542 to 0.628 (by 15.9%), on the right‐side chin from 0.655 to 0.723 (by 10.4%), and on the left side of the chin from 0.647 to 0.745 (by 15.1%).

The comparison of treatment‐induced changes (T1–T0) in the Q2 parameter between the left and right sides of the face showed no statistically significant differences for the cheeks (*p* = 0.797) or chin (*p* = 0.797). However, for the forehead, the differences between the two sides were statistically significant (*p* = 0.033).

The analysis of skin biomechanical parameters following the application of the tested preparations indicates a marked and statistically significant enhancement in skin elasticity, viscoelasticity, and residual deformation for both concentrations of RAL. The R5 and R7 parameters, widely validated in scientific literature, exhibited statistically significant increases across all tested facial areas on both sides.

## 4 Discussion

Research by Brown et al. supports the concept that RAL significantly enhances skin attributes crucial for minimizing visible signs of aging, such as wrinkles and fine lines [10]. The study specifically highlights how RAL notably improves skin elasticity and hydration, leading to better skin texture and a reduction in wrinkle depth [10]. These outcomes are particularly pronounced with higher RAL concentrations, aligning closely with the findings presented in this research.

Moreover, the biochemical mechanisms through which RAL influences skin condition are well‐documented. Retinoids are known to accelerate cellular turnover and increase collagen production, two critical factors in skin rejuvenation [11]. RAL, as the immediate precursor of retinoic acid (RA) in the vitamin A metabolic pathway, exhibits potent antiaging activity through the activation of multiple molecular mechanisms in the skin. Once converted to retinoic acid, RAL binds to nuclear retinoid receptors RAR (Retinoic Acid Receptors) and RXR (Retinoid X Receptors), which form heterodimers and interact with retinoic acid response elements (RAREs) in DNA. This interaction leads to transcriptional regulation of genes involved in cell proliferation and differentiation, and most importantly, in the synthesis of type I and III collagen and the remodeling of the dermal extracellular matrix. Retinoids also inhibit the expression of the transcription factor AP‐1, which stimulates the production of collagenases and other matrix‐degrading enzymes, thereby limiting collagen breakdown and enhancing its synthesis. Additionally, retinaldehyde exerts epigenetic effects, promoting renewal of the stratum corneum and reorganization of the epidermal layers [5, 12, 13]. Cordero et al. demonstrate that the combination of RAL and hyaluronic acid fragments (HAFi) effectively improves wrinkles, skin elasticity, hyperpigmentation, and ptosis. The study utilized optical profilometry to quantitatively assess changes in skin surface [14]. These properties support the improvements in biophysical and biomechanical parameters observed in our study.

The observation that both 0.05% and 0.1% RAL concentrations significantly improved dermal density, whereas a significant reduction in wrinkles was achieved only with the higher 0.1% dose, highlights the presence of distinct threshold effects for various antiaging outcomes. This finding is consonant with controlled studies. Kwon et al. [15] demonstrated that both concentrations (0.1% and 0.05%) improved skin texture, barrier function, and hydration after 3 months of use, but only the 0.1% RAL group achieved statistically significant improvement in melanin index—a proxy for deeper photodamage and advanced aging features. In the same study, the application of a cream containing either 0.1% or 0.05% retinaldehyde twice daily for 3 months resulted in a reduction of wrinkles in the eye area, with a slightly greater effect observed in the group using the higher concentration (1.8% vs. 1.5%). Additionally, a more pronounced improvement in skin texture was noted among individuals using the 0.1% RAL formulation (13.7% vs. 12.6%), further supporting the notion of dose‐dependent efficacy in certain antiaging parameters. The dose‐dependent effect of RAL on wrinkle reduction represents a notable finding, with statistically significant improvements observed in key areas such as the chin and forehead. These findings are consistent with the established role of retinoids in promoting cell turnover and alleviating signs of photoaging. Sum et al. note that RAL can mitigate the effects of photoaging by upregulating collagen synthesis [16]. This is particularly relevant for cosmetic formulations aimed at older adults or individuals with significant sun exposure. The observed trend toward reduced wrinkle length and area underscores the potential of retinol‐based formulations as effective antiaging agents, warranting further investigation into optimal dosing regimens [10, 14].

The comparative effectiveness of RAL against other retinoids has also been explored. Studies suggest that RAL may provide a more favorable efficacy and tolerability profile, making it suitable for sensitive skin types without compromising on results [17].

In our study, we employed noninvasive techniques, such as high‐frequency ultrasonography and Cutometer measurements, to evaluate changes in skin elasticity and collagen density. In contrast, research by Sum et al. and Kong et al. employed invasive methods, involving skin biopsies to assess skin properties [16, 18]. Noninvasive methods can provide significant insights into skin health without the discomfort and potential complications associated with invasive procedures, making them more suitable for repeated and routine clinical use.

HFUS is a noninvasive method used in medicine and dermatology for assessing skin lesions, aesthetic concerns, and potentially serving as an alternative to biopsy. Currently, noninvasive techniques such as HFUS can be employed to detect and thus assist in the diagnosis of late‐stage metastatic cancer, particularly when secondary tumors have grown sufficiently large. In this context, HFUS demonstrates potential for identifying local superficial metastases of melanoma, providing a valuable tool for early detection and treatment planning [19].

The Antera 3D camera has emerged as a significant tool in dermatological research, enabling precise 3D analysis of the skin's surface. Its ability to provide detailed, high‐resolution images is particularly valuable for assessing changes in skin topography, such as wrinkles, texture, and pigmentation, which are crucial for both clinical evaluations and cosmetic product testing. In the pilot trial conducted by Puviani and Milani, the Antera 3D camera was crucial for assessing the effectiveness of a combined treatment involving a piroxicam cream and a retinoic/glycolic gel on actinic keratosis lesions [20]. This study demonstrated significant reductions in lesion thickness and erythema scores, showcasing the camera's ability to deliver precise, real‐time measurements of treatment outcomes. The use of the Antera 3D camera in this context underscores its value in dermatological research, enabling detailed, noninvasive monitoring of skin condition changes throughout treatment.

In the context of dermatological research, the Cutometer has proven to be a valuable tool for assessing the biomechanical properties of the skin, particularly skin elasticity and firmness. The device operates on the principle of suction, measuring how the skin deforms and returns to its original shape, thereby allowing researchers to quantify skin elasticity and other related parameters effectively. Studies have utilized the Cutometer to evaluate the effectiveness of various skin treatments, underscoring its significance in both clinical and cosmetic dermatology [21, 22]. However, its application in assessing the impact of RAL on skin properties represents an innovative aspect of this research. This novel approach could provide deeper insights into how RAL influences skin biomechanics, offering a unique contribution to dermatological studies.

## 5 Limitations

Several limitations should be acknowledged in this study. First, we included only female participants aged 30–58 years, which limits the generalizability of our findings to the male population and to younger or older age groups. Additionally, our study population consisted exclusively of Caucasian participants with Fitzpatrick skin types II and III, which limits the applicability of results to other ethnic groups and skin phototypes. Future studies should include male subjects, broader age ranges including elderly populations, and diverse ethnic groups with different skin types to determine if similar beneficial effects of retinaldehyde occur across different demographics. Hormonal differences and variations in skin structure between different populations may influence treatment outcomes and should be considered in future research. Another limitation is that the study was single‐blinded (participants only) rather than double‐blinded, and we did not randomize the side allocation (all participants used 0.1% on left side and 0.05% on right side). However, the risk of bias was minimized by using objective instrumental measurements and standardized protocols. Finally, we used proprietary commercial preparations, so detailed formulation composition and preparation methods were not available for analysis.

## 6 Conclusions

Overall, the tested preparations were well tolerated by participants throughout the 24‐week period of use. Only one out of 56 volunteers experienced skin irritation as an adverse event. Among the study participants, 85% reported visible improvement in their skin condition after retinol treatment, with 82% expressing willingness to continue using the tested preparations. The tested preparations did not induce hyperkeratosis, cause stratum corneum thinning, or alter epidermal thickness in study subjects with normal keratinization processes, as validated by ultrasound assessments. High‐frequency ultrasound studies revealed a significant increase in skin density across all examined areas, indicative of the production of new collagen fibers and enhanced skin firmness post‐preparation use. Cutometric testing further confirmed notable improvement in skin biomechanical parameters, suggesting enhanced skin tension and elasticity. Furthermore, a greater increase in skin density was observed after 24 weeks compared with 12 weeks of using the preparations, underscoring the benefits of long‐term use. Although both concentrations were effective, the 0.1% concentration demonstrated a statistically significant reduction in wrinkles in specific areas, which was not observed with the 0.05% concentration.

## Funding

This study was funded by the Medical University of Silesia (Grant Numbers: BNW‐1‐025/N/4/K) and PH DOCTOR N&D Wiśniewscy S.K.A.

## Disclosure

One of the co‐authors is an employee of PH DOCTOR N&D Wiśniewscy S.K.A., the company that provided the tested products and partially funded the study. The remaining authors are affiliated with the Medical University of Silesia and received institutional compensation for their participation in the project. The authors declare that the study was conducted independently and that the sponsor had no influence on data collection, data interpretation, or the preparation of this manuscript.

## Ethics Statement

The study was conducted in accordance with the Declaration of Helsinki, and approved by the Bioethics Committee of the Medical University of Silesia (PCN/CBN/0022/KB1/27/III/16/17/21). All participants provided written informed consent for study participation and publication of their photographs.

## Data Availability

The data that support the findings of this study are available from the corresponding author upon reasonable request.
